# Docetaxel Combined with Thymoquinone Induces Apoptosis in Prostate Cancer Cells via Inhibition of the PI3K/AKT Signaling Pathway

**DOI:** 10.3390/cancers11091390

**Published:** 2019-09-18

**Authors:** Santosh Kumar Singh, Tejumola Apata, Jennifer B. Gordetsky, Rajesh Singh

**Affiliations:** 1 Department of Microbiology, Biochemistry and Immunology, Morehouse School of Medicine, Atlanta, GA 30310, USA; sksingh@msm.edu (S.K.S.); apatatejumola@gmail.com (T.A.); 2 Departments of Pathology and Urology, Vanderbilt University Medical Center, Nashville, TN 37232, USA; Jennifer.b.gordetsky@vumc.org

**Keywords:** thymoquinone, docetaxel, PI3K/AKT, apoptosis, prostate cancer

## Abstract

Toxicity and the development of resistance by cancer cells are impediments for docetaxel (DTX), a primary drug for treating prostate cancer (PCa). Since the combination of DTX with natural compounds can increase its effectiveness by reducing its toxic concentrations, we evaluated a combination of thymoquinone (TQ) with DTX and determined its cytotoxicity against PCa cells (DU145 and C4-2B). This combination, in a concentration-dependent manner, resulted in synergistic cytotoxicity and apoptosis in comparison to either DTX or TQ alone. In addition, inhibition of cell survival pathways by PI3K/AKT inhibitors conferred sensitivity of DU145 and C4-2B cells to the combination as compared to the individual drugs. Moreover, the combined drugs (DTX+TQ) with inhibitors of PI3K/AKT increased the expression of pro-apoptotic markers (BAX and BID) along with caspase-3, PARP and decreased expression of the anti-apoptotic marker, BCL-XL. These data show that, for PCa cells, the cytotoxic effect of the DTX and TQ combination correlates with a block of the PI3K/AKT signaling pathway. These findings indicate that the combination of DTX and TQ, by blocking of the PI3K/AKT pathway, will improve the survival rate and quality of life of PCa patients.

## 1. Introduction

Prostate cancer (PCa) is common in men, causing 250,000 deaths each year [1]. In 90% of cases, PCa is diagnosed as organ-confined or locally advanced, based on clinical stage and levels of prostate-specific antigen. Although PCa can be diagnosed at early stages, some patients develop metastases after local treatment with surgery/radiotherapy [2]. With these considerations, decisions are made whether to opt for active surveillance, or definitive therapy [3]. With the currently available agents, responses are transient; however, secondary hormonal therapy is an option for some patients. PCa that has advanced despite the achievement of castrate levels of androgens are considered as castration-resistant (CRPCs). High levels of prostate-specific antigen, substantive pain, and bone and lymph node metastases are associated with patient morbidity [4].

New drugs, drug sequences, and drug combinations, recently used for treatment of PCa, have improved the outcomes for PCa patients. Among these is taxane-based chemotherapy [3]. Until 2010, the taxol-derived drug, docetaxel (DTX), which prevents microtubule de-polymerization and mitotic division, was the standard chemotherapeutic treatment for PCa patients [5]. DTX combined with androgen deprivation therapy improves the outcomes for men with metastases at the first presentation [6,7,8]. Despite being effective in treating PCa, DTX resistance due to overexpression of ABC transporter efflux proteins, which limit the intracellular concentrations of the drug, is an impediment for treating PCa [9]. In addition, DTX treatment induces adverse events, including febrile neutropenia, fatigue, pneumonitis, infusion reactions, fluid retention, cutaneous and nail toxicity, gastrointestinal complications, and neuropathies [10,11]. To circumvent the negative effects and benefit from the useful effects of DTX, a combination of DTX with natural anticancer agents is a relevant approach for the treatment of PCa [12,13].

Thymoquinone (TQ), a phytochemical commonly found in black cumin (*Nigella sativa*), has a long history of medicinal use [14,15]. Both oil and seeds from *Nigella* plants have anticancer, antidiabetic, antihypertensive, and antimicrobial effects [16,17]. In cancer research, TQ shows promising activity in cell culture and animal models [18], and it has anti-proliferative effects for ovary, colon, larynx, breast, and lung cancer cells and for myeloblastic leukemia and osteosarcoma cells [19]. In treated cells, TQ induces apoptosis, chromatin condensation, DNA fragmentation, and translocation of phosphatidylserine across the plasma membrane [18]. In addition to its anti-cancer properties, TQ strengthens the immune system, protects normal cells from oxidative damage, and prevents toxic side effects [19].

In PCa, various growth and survival, promoting pathways interact. The role of phosphatidylinositol-4,5-bisphosphate 3-kinase (PI3K)/protein kinase B (AKT) is being studied, and treatments involving a single inhibitor or a combination of agents who interfere with the androgen receptor are being investigated in clinical studies [20]. For PCa, the activated PI3K/AKT pathway is associated with progression, resistance, and metastasis of cancer cells. Aggressive cancers are characterized by the survival, growth, metabolic, and metastatic functions signaled through this pathway. High grade and progression of PCa correlate with its activation [21]. Although inhibitors of this pathway show antitumor activity in animals [22,23], initial clinical studies of these agents have shown only limited efficacy [3]. Thus, there is a need for developing new agents to target this pathway in PCa. In the present investigation, we used DTX and TQ in combination for treating PCa cells. By activating pro-apoptotic proteins, the combination showed a cytotoxic effect on PCa cells. In addition, in the presence of PI3K/AKT inhibitors, the combination of DTX and TQ activated apoptosis and inhibited expression of an anti-apoptotic gene. 

## 2. Results

### 2.1. Effects of TQ, DTX, and their Combination on Cell Viability, Proliferation, and Cytotoxicity of PCa Cells

**Figure 1:**
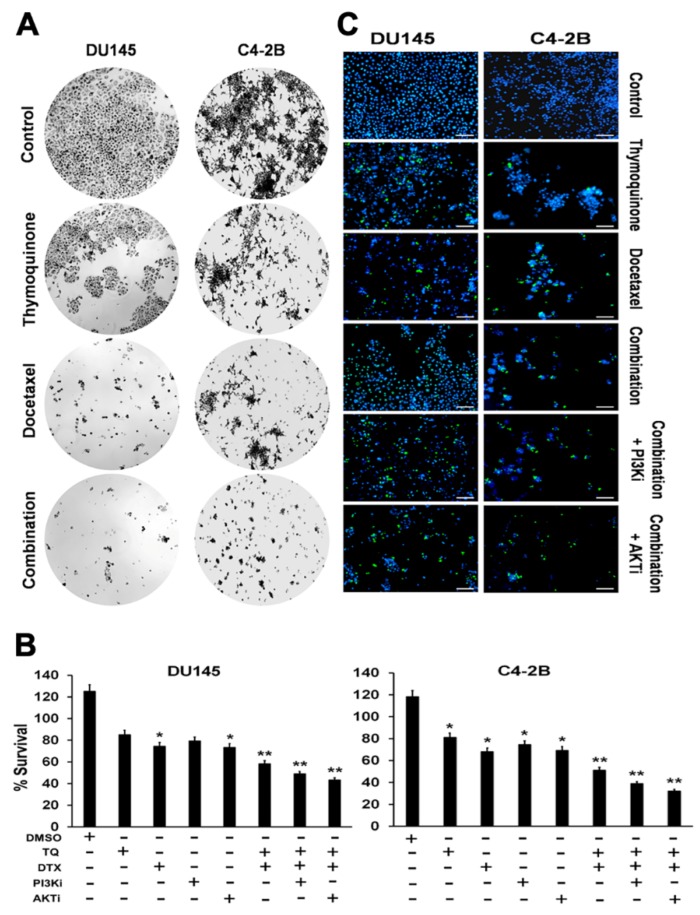
Effect of TQ and DTX on cell viability and cytotoxicity to PCa cells. PCa cells were treated with sequential concentrations of TQ, DTX, or a combination of TQ and DTX for 48 h, and viability was measured by MTT assays for (**A**) DU145 and C4-2B cells. DU145 cells were treated with TQ (60 µM), DTX (20 nM), or their combination (TQ+DTX, 50 µM + 10 nM); C4-2B cells were treated with TQ (54 µM), DTX (20 nM), or their combination (TQ + DTX, 35 µM + 10 nM) for 48 h. (**B**) shows the percentage survival of DU145 and C4-2B cells after treatment with TQ, DTX, PI3Ki, AKTi or their combination with or without PI3Ki (1.4 µM) and AKTi (625 nM) for 48 h. Sign “+ or −“ represents the presence or absence of the drugs. Data are presented as means ± standard error of the means of at least three independent experiments and were analyzed by an unpaired t-test. ** and * indicate *p* values of ≤0.01 and 0.05, respectively. (**C**) shows the DU145 and C4-2B cells treated with TQ, DTX, or their combination with or without PI3Ki and AKTi for 48 h and were processed for immunofluorescent staining. More live nuclei (blue) were found in the control group compared to any of the treatment groups. More dead nuclei (green) were found in those treated with the combination in presence or absence of PI3Ki and AKTi compared to treatment with individual drugs. Nuclei of live and dead cells, detected by DAPI staining and a GFP filter, appear as blue or green, respectively.

To determine the therapeutic potentials of TQ, DTX, and their combination, viability assays were performed for C4-2B and DU145 cells. In addition, the toxicity of TQ and DTX combined was determined in the presence of the PI3Ki and AKTi. Three-time points (24, 48, and 72 h) were used to determine the IC50 values for each individual drug and the combined drugs. Among the three time points, a concentration-dependent apoptotic response was found at the 48-h time point. DU-145 cells had IC50 values of 60 µM for TQ, 20 nM for DTX, and 50 µM + 10 nM for their combination. In comparison, C4-2B cells had IC50 values for TQ, DTX, and their combination of 54 µM, 20 nM, and 35µM + 10 nM, respectively (Figure 1A). The combination index (CI) value was found to be 0.41 (CI = 0.41) and 0.32 (CI = 0.32) in DU145 and C4-2B cells when treated with combined drug (TQ + DTX). These results showed that DU-145 cells had higher tolerance to TQ compared to C4-2B cells, in contrast the synergistic effect of combined drug was found more efficient in C4-2B compared to DU145 cells. Although TQ and DTX were individually toxic to PCa cells, their combination had a higher apoptosis-inducing effect, with lower concentrations of both agents being required. In addition to the combined effect of TQ and DTX against PCa cells, their action on cell survival was assessed in the presence of PI3Ki (1.4 µM) and AKTi (625 nM) using a survival assay. There was lower survival of PCa cells with a combination of TQ and DTX along with PI3Ki and AKTi (Figure 1B). There were similar results with a cell viability test in which stained cells were represented by blue and green colors for nuclei of live and dead cells, respectively. More dead cells were evident when they were treated with PI3Ki and AKTi combined with TQ and DTX compared to individual drugs or their combination of TQ and DTX without the inhibitors (Figure 1C). C4-2B cells had more dead nuclei compared to DU145 cells, showing some drug tolerance of DU145 cells. These results suggest that, for PCa cells, TQ synergizes with DTX and enhances the efficacy of DTX.

### 2.2. The DTX and TQ Combination Induces Apoptosis in PCa Cells

**Figure 2:**
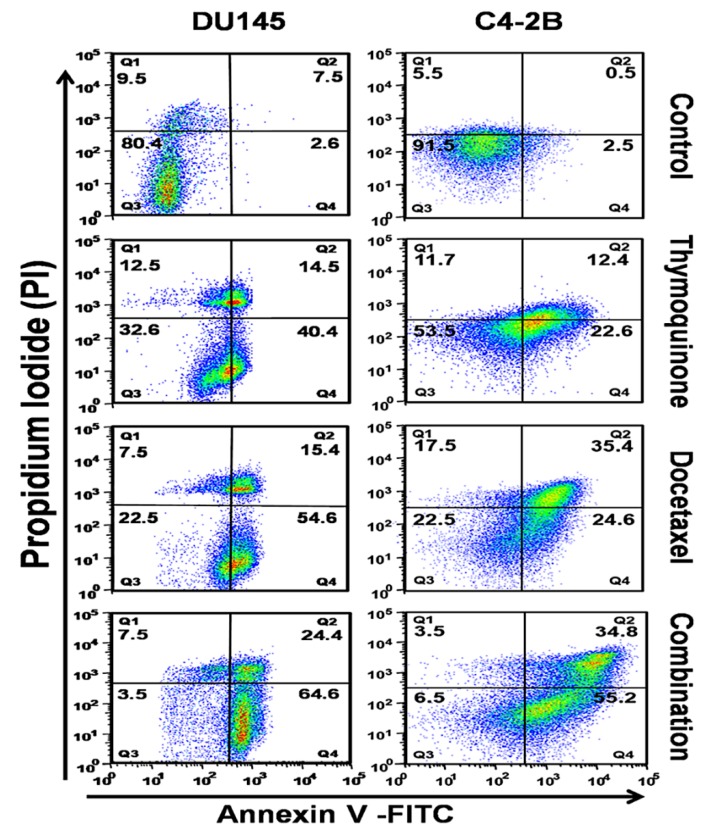
TQ induces DTX-mediated apoptosis in PCa cells. DU145 and C4-2B cells were treated with TQ, DTX, or their combination for 48 h, and apoptosis was assessed by staining with Annexin V-FITC and PI followed by flow cytometry. The percentages of early (Annexin(+)/(PI(−), late (Annexin(+)/(PI(+), necrotic (Annexin(−)/(PI(+), and viable (Annexin(−)/(PI(−) cells are shown in bold numbers in Q4, Q2, Q1, and Q3, respectively. For both DU145 and C4-2B cells, the combination of TQ + DTX showed high numbers of apoptotic cells in the (early) or (late) quadrants compared to individual drugs alone. Data shown are the means ± SEM from three independent experiments.

To investigate the extent of apoptotic induction by the TQ and DTX combination in comparison to DTX or TQ alone, we evaluated the cell death effect on DU145 and C4-2B cells by TQ alone, DTX alone, or in combination for 48 h. After treatment, cells were stained by using an apoptosis kit (with Annexin V-FITC and PI) and analyzed by flow cytometry. Quadrants Q1–Q4 represent necrotic, late apoptotic, viable, and early apoptotic cells, respectively (Figure 2). For DU145 cells treated with TQ alone, DTX alone, or their combination, values for the total (early and late) apoptotic cells were 54.9%, 70%, and 89%, respectively, compared to the control (10.1%). However, C4-2B cells had values for early and late apoptotic cells for TQ (35%), DTX (60%), and the combination (90%), respectively. The TQ + DTX combination increased the extent of apoptosis compared with either drug alone. These results confirm the cytotoxicity and efficacy of TQ when given in combination with DTX to treat PCa cells. 

### 2.3. Effect of PI3K/AKT Inhibition on PCa Cells

**Figure 3:**
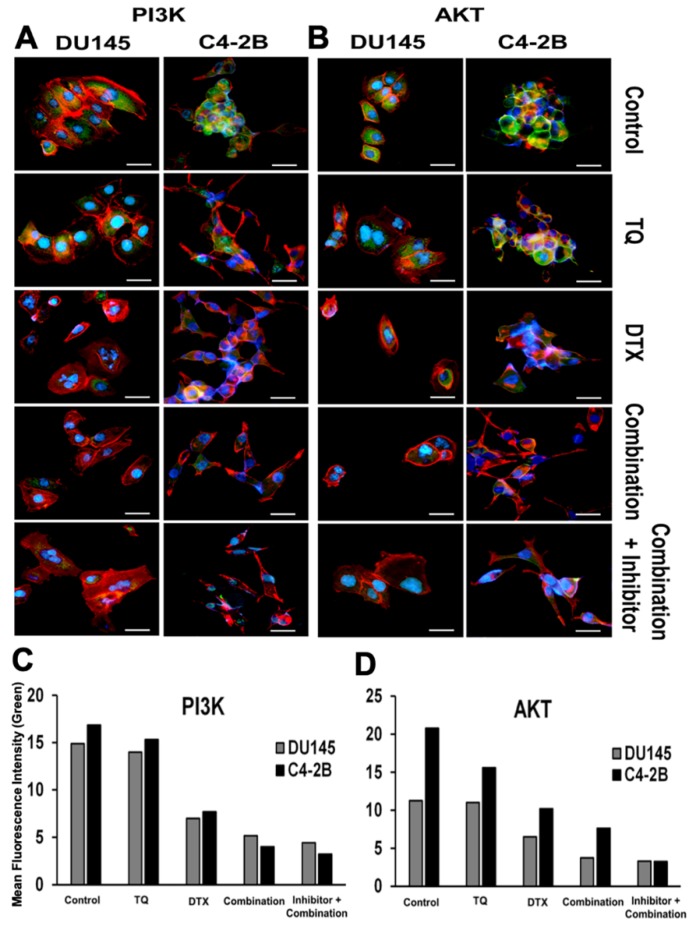
Immunofluorescent analysis of PI3K/AKT expression in PCa cells. DU145 and C4-2B cells, cultured overnight, were treated with TQ, DTX, or their combination with or without PI3Ki and AKTi for 48 h and were processed for immunofluorescent staining. For (**A**) PI3K, and (**B**) Akt expression, cells were stained with primary and Alexa Fluor 488-conjugated secondary antibodies and examined with a fluorescent microscope. (**C**,**D**) represent the mean fluorescence intensity of PI3K or AKT (green) for DU145 and C4-2B cells respectively. Green indicates the expression of PI3K or Akt; red indicates Phalloidin staining. Nuclei were stained with DAPI, and images were captured at 40× magnification.

To determine if the PI3K/AKT pathway was involved in the proliferation and progression of PCa cells, we evaluated its expression in DU145 and C4-2B cells. Based on their IC50 values, PCa cells were treated with TQ or DTX or with the combination. Upon treatment, cells were exposed to PI3Ki (1.4 µM) and AKTi (625 nM) for 48 h with a combination of TQ + DTX. DU145 cells showed a lower expression of PI3K/AKT when treated with the combination in presence of inhibitors compared to control (untreated), individual drugs, or their combination. Similarly, in C4-2B cells, the expression was low upon blocking of PI3K/AKT (Figure 3A,B). These results suggesting that reduced phosphorylation of PI3K/AKT inhibits the downstream signaling effectors that lead to cell apoptosis.

### 2.4. The TQ and DTX Combination induced Modulation of Pro- and Anti-Apoptotic Molecules via Inhibition of the PI3K/AKT Pathway in PCa Cells

**Figure 4:**
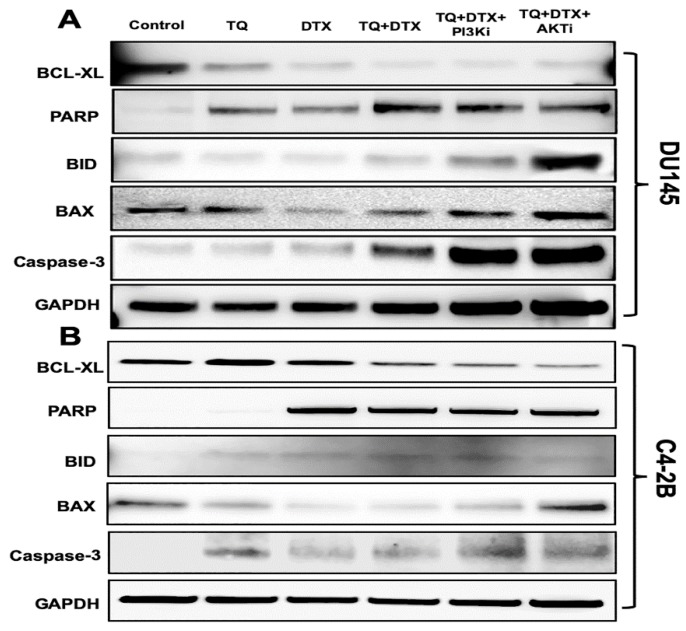
TQ in combination with DTX induces the expression of pro- and anti-apoptotic markers in PCa cells. Immunoblots of (**A**) DU145 and (**B**) C4-2B cells showing the expression of pro- and anti-apoptotic genes after treatment with TQ, DTX, or their combination in the presence or absence of PI3Ki and AKTi inhibitor. After 48 h of treatment with the combination, the pro-apoptotic genes, BAX and BID, and caspase-3 and PARP were upregulated relative to effects of the individual drugs. However, for both cells, the anti-apoptotic gene, BCL-XL, was downregulated by the combination. As an internal standard for equal loading, blots were probed with a GAPDH antibody.

Cellular signals regulate cancer cell proliferation and apoptosis, and inhibition of these signals can be helpful in eliminating cancer [24]. Considering these facts, we validated the expression of apoptosis-specific genes at the protein level by treating DU145 and C4-2B cells with TQ, DTX, or their combination in the presence and absence of PI3Ki and AKTi. The expressions of pro-apoptotic genes (BID and BAX) were upregulated in DU145 cells treated with the combination (TQ + DTX, 50 μM + 10 nM) and in the presence of PI3Ki (1.4 µM) and AKTi (625 nM) for 48 h; in turn, the expression of BCL-XL was downregulated (Figure 4A). Although the individual drugs were effective, their action on apoptotic genes were minimal. Subsequently, C4-2B cells were treated with TQ (54 µM), DTX (20 nM), or their combination (TQ+DTX, 35 µM +10 nM) with or without PI3Ki (1.4 µM) and AKTi (625 nM) to monitor cell death (Figure 4B). The expressions of pro-apoptotic proteins (BID and BAX) were upregulated, and the anti-apoptotic protein, BCL-XL, was downregulated upon treatment of cells with the inhibitors, confirming the effectiveness of TQ and DTX in PCa cells. In addition to effects on pro- and anti-apoptotic genes, there was, in both cell lines at 48 h after treatment, upregulation of pro-caspase 3. Moreover, in respect to DNA damage caused by the combination with inhibitors, poly(ADP-ribose) polymerase (PARP)-1 expression was elevated compared to expression in the control cells, and in cells exposed to the individual drugs (not in the case of DTX alone in C4-2B cells). The protein band intensity for DU145 and C4-2B cells are shown in Figures S1 and S2, respectively. Thus, the combination of TQ and DTX promoted cell death compared to TQ or DTX alone. 

### 2.5. The TQ and DTX Combination Regulated Gene Expression at the mRNA Level in PCa Cells

**Figure 5:**
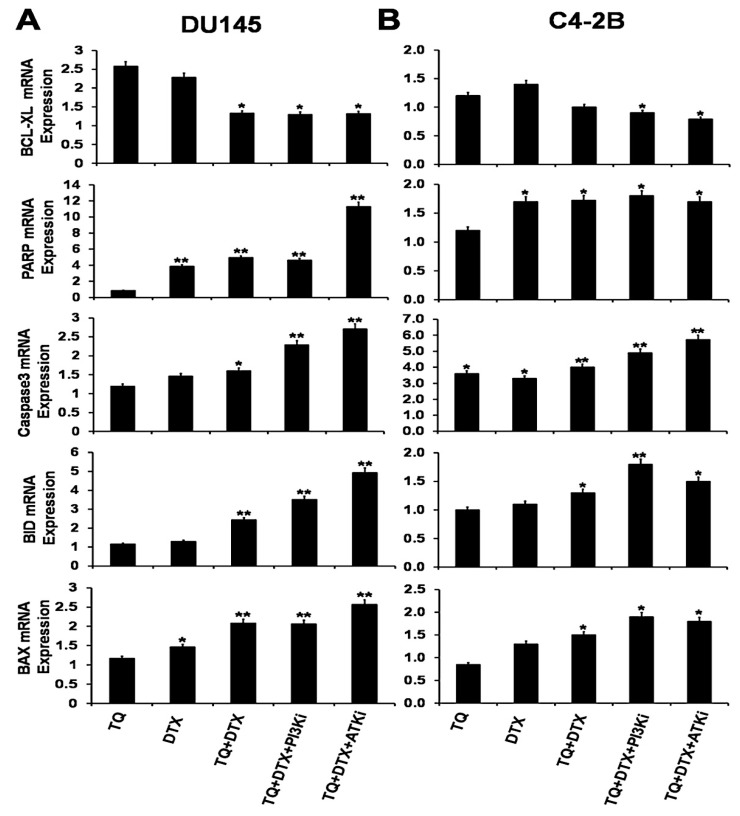
mRNA expression of pro- and anti-apoptotic genes in PCa cells. (**A**) DU145 and (**B**) C4-2B cells were treated with various concentrations of TQ, DTX, or their combination with or without PI3Ki and AKTi for 48 h and quantified by RT-PCR. The expression levels of pro-apoptotic factors (BAX, and BID), PARP, caspase 3, and the anti- apoptotic factor (BCL-XL) are presented as fold changes relative to the control cells. Data were normalized to the levels of expression of the housekeeping gene (18 S), and the experiments were repeated three times. Data are presented as means ± SEM; asterisks indicate significance as determined by Student *t*-tests. * and ** indicates *p* value ≤ 0.01 and 0.05, respectively.

Inhibition of the PI3K/AKT signaling pathway in cancer cells is effective in inducing apoptosis [25]. Combinations of drugs have been investigated for tumor cells prevention and treatment. However, since there is no established chemotherapeutic cure for PCa patients, a new strategy or drug target for PCa treatment is needed. Here, we studied the expression of genes in PCa cells in response to TQ, a natural compound, and DTX and to blocking of the PI3K/AKT signaling pathways. For DU145 cells, pro-apoptotic (BAX and BID genes) and PARP expression were upregulated after blocking the PI3K/AKT pathways with PI3Ki and AKTi compared to the effects of the single drugs alone or in combination (Figure 5A). There was an increase in the apoptotic gene, caspase 3, after 48 h of treatment with TQ, DTX, or their combination. To assess the anti-apoptotic genes in DU145 cells, we tested the marker, BCL-XL; there was no appreciable difference for their combination treatment with or without inhibitors.

Further, to validate the pro- and anti-apoptotic markers in C4-2B cells, these cells were treated for 48 h based on their IC50 values. The pro-apoptotic BAX and BID genes at their mRNA levels were elevated in the presence of blocking agents (Figure 5B). In line with that, the expression of caspase 3 was upregulated when the pathways were blocked with PI3Ki and AKTi inhibitors. PARP expression was upregulated, but there was no appreciable difference between the effects of the combination and with the inhibitors. BCL-XL was suppressed by the combination. Together, these observations suggest that, for their combination of TQ and DTX, the PI3k/AKT signaling cascade is blocked, which causes apoptosis in PCa cells. 

## 3. Discussion

Despite improvements in hormone therapy, many men with PCa develop metastatic CRPC and require chemotherapy. DTX is widely used for treating PCa and is a primary agent for treating other cancers. To reduce the side effects of DTX and improve its efficacy, it has been administered with other drugs. Several epithelial cancers have been treated with combinations containing taxane-based chemotherapeutic agents, but PCa remains as an exception [26]. Although a recent study suggested that histone deacetylase inhibitors, in combination with DTX, enhance its anti-proliferative effect in CRPC cells [27], previous efforts in combination treatment show no additional benefit or increased side effects such as thromboembolic events [1]. In addition, phase III trials in treating CRPC have failed to improve on DTX efficacy [1]. Hence, combination strategies that improve the outcomes for PCa are needed. 

With a long history of use of natural products/compounds from plants, microorganisms and fungi against human diseases, they have now been tested against cancer, and some have shown promising results [28]. Among these is TQ, which, by acting on various pathways, shows promising effects for humans [19]. Although, several randomized control trials (RCTs) have been investigated the therapeutic effects of TQ in the prevention and the treatment of various diseases in humans [29]. In the present effort, we investigated the combination of TQ with DTX against PCa cell viability, proliferation, and cytotoxicity. Apoptosis, a physiological form of cell death and a pathway for regulating homeostasis and morphogenesis of cells, is associated with various diseases, in particular, cancer [30]. Apoptosis is described as the organized collapse of cells by membrane blebbing, cell shrinkage, chromatin condensation, and DNA fragmentation [31]. Studies on the mechanism of action of TQ show that it induces apoptosis of cancer cells [31]. Taxanes have a broad spectrum of activity and induce apoptosis of cancer cells in varying degrees [32]. In the present investigation, analysis of changes in apoptosis using Annexin V in the multidomain spectrum of flow cytometry showed that TQ and DTX individually cause apoptosis of PCa cells. However, the extent of apoptosis was higher when these anticancer agents were combined in low concentrations. These results show that TQ can reduce the side effects of DTX and lead to an enhancement of apoptosis of cancer cells. 

DTX induces apoptosis in cancer cells, as demonstrated by associated changes in the mitochondrial membrane potential and overexpression of BCL2 [32]. Although the activation of caspase 8 and BID seems to be a late event, apoptosis is induced by the activation of caspase 2 [32]. For PCa cells, TQ activates apoptosis-inducing factor-1 and the DNA damage-inducible gene, GADD45 alpha, and down-regulates the expression of Bcl2, Bcl2L1, BAG-1, Bcl2A1, and BID proteins [19]. The present investigation showed induced expression, at mRNA and protein levels, of pro-apoptotic genes and downregulation of anti-apoptotic genes. As individual compounds, TQ and DTX did not have a profound effect on the expression of BCL-XL, but the combination caused a pronounced decrease. For their combination, there was a reverse trend for pro-apoptotic genes, BAX and BID, as well as high expression of pro-caspase 3 protein. These results show that the combination of the two agents, acting by two mechanisms (inhibiting PI3K/AKT pathway and inducing apoptosis), facilitate apoptosis and that the combination can be used to treat PCa. 

PI3K/AKT signaling appears to be necessary for PCa cell survival and proliferation [33]. Due to the aggressive nature of the PI3K/AKT pathway and its frequent presence in metastatic PCa, this pathway has been used as a biomarker for progressive PCa [21]. To combat PCa, combination therapy with a PI3K/AKT inhibitor can be regarded as horizontal blockade [34]. In breast [35] and gastric cancers [36], TQ inhibits the PI3K/AKT pathway by downregulating the phosphorylation of AKT and upregulating PTEN. In addition, DTX is a candidate for inhibition of the PI3K/AKT pathway. In the current study, we interrogated the effect of PI3K/AKT inhibitors and DTX and TQ individually in affecting the PI3K/AKT pathway and compared the effects with the combination of DTX and TQ and along with PI3K/AKT inhibitors. The combination inhibits the PI3K/AKT pathway, which induces death of PCa cells.

## 4. Materials and Methods

### 4.1. Cell Culture

The PCa cell lines, DU-145 (HTB-81) and C4-2B (CRL-3315), were procured from the American Tissue Culture Collection (ATCC, Manassas, VA, USA). DU-145 cells were grown in Eagle’s Minimum Essential Media (EMEM) supplemented with 10% fetal bovine serum (FBS), and 10,000 U/mL penicillin/10,000 μg/mL streptomycin antibiotic solution (Fisher Scientific, Pittsburgh, PA, USA). C4-2B cells were maintained in DMEM plus F12 Medium (Fisher Scientific, Pittsburgh, PA, USA) supplemented with 10% heat-inactivated FBS, insulin, triiodo-L-thyronine, transferrin, D-biotin, adenine (Millipore Sigma, St. Louis, MO, USA), and 10,000 U/mL penicillin/10,000 μg/mL streptomycin antibiotic solution (Fisher Scientific, Pittsburgh, PA, USA). Both cell lines were maintained in an incubator at 37 °C and with 5% CO_2_.

### 4.2. Cell Proliferation Assay

Growing cells were trypsinized (0.25% Trypsin-EDTA, Fisher Scientific, Pittsburgh, PA, USA) and seeded in 96-well plates at 10,000 cells/well. Cells were treated with DTX (0, 5, 10, or 50 nM), TQ (0, 5, 10, 15, 20, 25, or 50 µM) (Fisher Scientific, Pittsburgh, PA, USA), and a combination of the drugs with an Akt inhibitor (AKTi) (0.5, 1, 2, 5, or 10 nM) and a PI3K inhibitor (PI3Ki, LY294002, 0.5, 1, 2, 5, or 10 µM) (Millipore Sigma, St. Louis, MO, USA). The cells were incubated for 24, 48, or 72 h at 37 °C in a 5% CO_2_ incubator. Following our previous protocol [12], 3-(4,5- dimethylthiazol-2yl) 2,5-diphenyltetrazolium bromide (MTT) 5 mg/mL), (Fisher Scientific, Pittsburgh, PA, USA) was added to each well. The cells were incubated at 37 °C for 3 h. The purple formazan crystals were dissolved in 100 µL of dimethyl sulfoxide, and the absorbance was measured at 570 nm with a spectrophotometer (Spectramax M5, Molecular Devices, Sunnyvale, CA, USA). Next, the IC50 (half-maximum inhibitory concentration) values were calculated for DU145 and C4-2B cells. To evaluate possible additive (0.9 < CI < 1.1), synergistic (CI < 0.9), or antagonistic effects (CI > 1.1), the combination index (CI) method was used [37], and the relative growth rates were calculated for both cell lines. 

### 4.3. Live/Dead Cell Assay

PCa cells (2 × 10^4^) were seeded in 48-well plates overnight and then treated with TQ, DTX, or a combination of TQ+DTX with or without PI3Ki and AKTi for 48 h. Cells were then washed with PBS and incubated with 2 drops/mL of cell viability imaging solution (Thermo Fisher Scientific, Carlsbad, CA, USA) for 15 min at 37 °C according to the manufacturer’s protocol. Fluorescence images were acquired after staining by using a DAPI, and FITC/GFP filter and an EVOS FL microscope (Thermo Fisher Scientific, Carlsbad, CA, USA).

### 4.4. Apoptosis Assay

DU145 and C4-2B cells were seeded in six-well plates and treated with TQ, DTX, or their combination for 48 h. Following our previous protocol [12], the cells were washed, harvested, and counted with a hemocytometer (Countess II FL, Life Technology, Carlsbad, CA, USA). Equal numbers (1 × 10^5^) of DU145 and C4-2B cells were washed twice with cell staining buffer (BioLegend, San Diego, CA, USA). Subsequently, cells were stained with FITC-Annexin V and propidium iodide (PI) according to the manufacturer’s instructions (BioLegend, San Diego, CA, USA). Further, cells were washed and suspended in Annexin V Binding Buffer. The apoptotic cells were processed with a Guava flow cytometer (EMD Millipore, Billerica, MA, USA) and analyzed with FlowJo software (FlowJo LLC, Ashland, OR, USA).

### 4.5. Immunofluorescence Assay

To determine expression of the intracellular signaling pathway (PI3K/Akt), DU145 and C4-2B cells were seeded in 48-well plates overnight at 37 °C and 5% CO_2_ and then treated with TQ, DTX, or with TQ+DTX with or without PI3Ki and AKTi for 48 h. The method for cell staining followed that described in our previous publication [38]. Briefly, cells were washed with cold PBS, fixed with 4% paraformaldehyde, and permeabilized with 0.05% saponin for 10 min. Next, the cells were washed, blocked with 3% bovine serum albumin, and stained with anti-PI3K and anti-Akt primary antibodies at 4 °C overnight, then incubated with FITC-conjugated fluorescent secondary antibody (Cell Signaling, Danvers, MA, USA) for 1 h. For visualization of the F-actin cytoskeleton, cells were stained with Phalloidin™ Red 594 solution (1:50, BioLegend, San Diego, CA, USA) for 20 min at room temperature. Cell nuclei were counterstained with DAPI (Invitrogen, Carlsbad, CA, USA). Immunofluorescence images were taken with a fluorescent microscope with DAPI, FITC/GFP, and RFP filters using an EVOS FL microscope (Thermo Fisher Scientific, Carlsbad, CA, USA).

### 4.6. Western Blot Analysis

To determine the expression of apoptotic markers, DU145 and C4-2B cells were treated with TQ, DTX, or their combination with or without PI3Ki and AKTi for 48 h. Cells were then harvested and lysed with RIPA buffer containing 1× protease and protease inhibitor cocktail (Thermo Fisher Scientific, Rockford, IL, USA) and centrifuged for 10 min at 10,000 rpm to collect the supernatants. Following our previous protocol for protein isolation [9], total protein was determined using BCA protein assay kits (Thermo Fisher Scientific, Rockford, IL, USA). Protein samples were prepared by using bromophenol blue dye and RIPA buffer and boiled for 10 min. Next, equal amounts of protein (30 µg) were fractionated on 4–12% polyacrylamide gels (Life Technologies, Carlsbad, CA, USA) and transferred to polyvinylidene fluoride membranes, which were blocked in 5% non-fat dry milk (Biorad, Hercules, CA, USA) for 30 min prepared with TBS-T (20 mM TRIS-HCl pH 7.6, 150 mM NaCl, 0.1% Tween 20), (Fisher Scientific, Pittsburgh, PA, USA). After blocking, blots were probed with primary antibodies overnight at 4 °C, followed by exposure to secondary antibodies (1:2000 dilution) for 1 h at room temperature. The primary antibodies, including those for pro-apoptotic (BAX and BID), anti-apoptotic (BCL-XL), and apoptotic (pro-caspase-3; BioLegend, San Diego, CA, USA) and poly[adenosine diphosphate]-ribose polymerase [PARP] factors, and secondary antibodies were procured from Cell Signaling Technology (Danvers, MA, USA). The resulting protein bands were visualized by chemiluminescence with Western detection reagent (Fisher Scientific, Pittsburgh, PA, USA), and images were acquired with an ImageQuant LAS4000 instrument (GE Healthcare-Biosciences, Pittsburgh, PA, USA).

### 4.7. RNA Isolation and Quantitative Reverse Transcription-Polymerase Chain Reaction (qRT-PCR)

PCa cells treated with TQ, DTX, or their combination and with PI3Ki and AKTi for 48 h were lysed with Trizol reagent (Invitrogen, Paisley, UK) and extracted following the standard protocol for RNA. Following our previous protocol [12], briefly, RNA was precipitated, suspended in nuclease-free water, and quantified. Total RNA (1.0 μg) and reverse transcription Supermix for RT-qPCR were used to generate cDNA following the manufacturer’s instructions (Bio-Rad, Hercules, CA, USA). Primers for pro-apoptotic (BAX and BID), anti-apoptotic (BCL-XL), and apoptotic factor (PARP and caspase-3) genes were synthesized with data from the National Center for Biotechnology Information (NCBI) gene bank and used for mRNA expression. 18 S primers (5′-GGCCCTGTAATTGGAATGAGTC-3′ and 5′-CCAAGATCCAACTACGAGCTT-3′) were used as an endogenous control. The following sequences of primers were used: BAX: 5′-AAACTGGT GCTCAAGGCCC-3′ and 5′-CTTCAGTGACTCGGCCAGG-3′; BID: 5′-AGCACAGTGCGGATTCTGTC-3′ and 5′-ACCGTTGTTGACCTCACAGT-3′; PARP: 5′-GCTTCAGCCTCCTTGCTACA-3′ and 5′-TTCGCCACTTCATCCACTCC-3′; caspase 3: 5′-CTCTGGTTTTCGGTGGGTGT-3′ and 5′-CGCTTCCATGTATGATCTTTGGTT-3′; and BCL-XL: 5′-CCTGCCTGCCTTTGCCTAA-3′ and 5′-TGGGCTCAACCAGTCCATTG-3. RT-PCR was performed using SYBR^®^ Green PCR master mix reagents (Bio-Rad, Hercules, CA, USA), and gene expression was analyzed by CFX-Manager software (CFX96 Real-Time System, Bio-Rad, Hercules, CA, USA). The experiments were repeated three times.

### 4.8. Statistical Analysis

All experimental data are presented as standard errors of means (±SEM) for at least three independent experiments. The level of significance was determined by one-way analysis of means (ANOVA), and *p*-values < 0.05 were considered statistically significant.

## 5. Conclusions

Despite improvements in anti-hormone therapy for PCa, many men develop CRPC and require chemotherapy. Although, for treating PCa, combinations containing DTX show potential in terms of tolerability and increased efficacy compared to DTX alone, no additional benefits have been observed. In the present investigation, we found that the combination of DTX with TQ had a greater cytotoxic effect against PCa cells, allowing decreases in the concentrations of both. Further, DTX and TQ were inducers of apoptosis; the extent of apoptosis was greater, even when they were used in lower concentrations. As many patients develop CRPCs, clinical trials are now evaluating the combination of PI3K/AKT inhibitors along with anticancer drugs. Thus, the combination of DTX and TQ in addition to PI3K/AKT inhibitors may improve the survival rate and quality of life of PCa patients.

## Supplementary Materials

The following are available online at https://www.mdpi.com/2072-6694/11/9/1390/s1, Figure S1: Densitometry Readings/intensity Ratio (DU145), Figure S2: Densitometry Readings/intensity Ratio (C42B). Western blot densitometry is available in supplementary materials (Figures S1 and S2).
