# Supplementary material for: Docetaxel Combined with Thymoquinone Induces Apoptosis in Prostate Cancer Cells via Inhibition of the PI3K/AKT Signaling Pathway
Source: Cancers (Basel). 2019 Sep 18;11(9):1390. doi: 10.3390/cancers11091390 (PMC6770702; doi:10.3390/cancers11091390)
Supplement: Supplementary file 1 [file cancers-11-01390-s001.pdf]

cancers

Supplementary Materials: Docetaxel Combined with Thymoquinone Induces Apoptosis in Prostate Cancer Cells via Inhibition of the PI3K/AKT Signaling Pathway

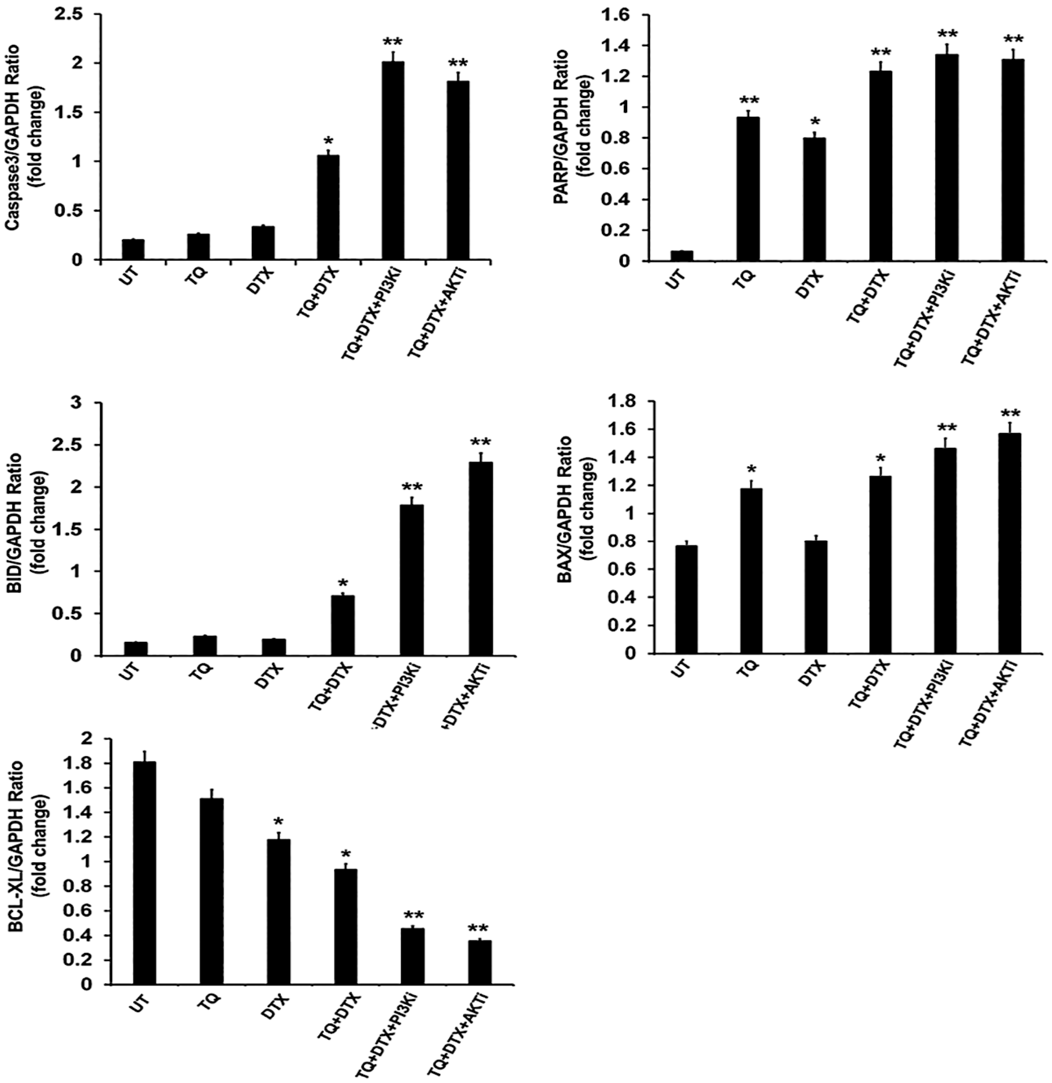

	Casapase 3/GAPDH	PARP/GAPDH	BID/GAPDH	BAX/GAPDH	BCL-XL/GAPDH
UT	0.199001332	0.061318242	0.152263648	0.76424767	1.805725699
TQ	0.258747493	0.930168635	0.229997771	1.1746527	1.510734715
DTX	0.336275146	0.795392602	0.192083063	0.801038287	1.175989617
TQ+DTX	1.058038099	1.229244555	0.703578423	1.262120942	0.933950507
TQ+DTX+PI3Ki	2.009865869	1.339430218	1.787163286	1.461478772	0.452887706
TQ+DTX+AKTi	1.81316001	1.306669809	2.288852227	1.566203158	0.354041951

(**i**) Densitometry Readings/intensity Ratio (DU145)

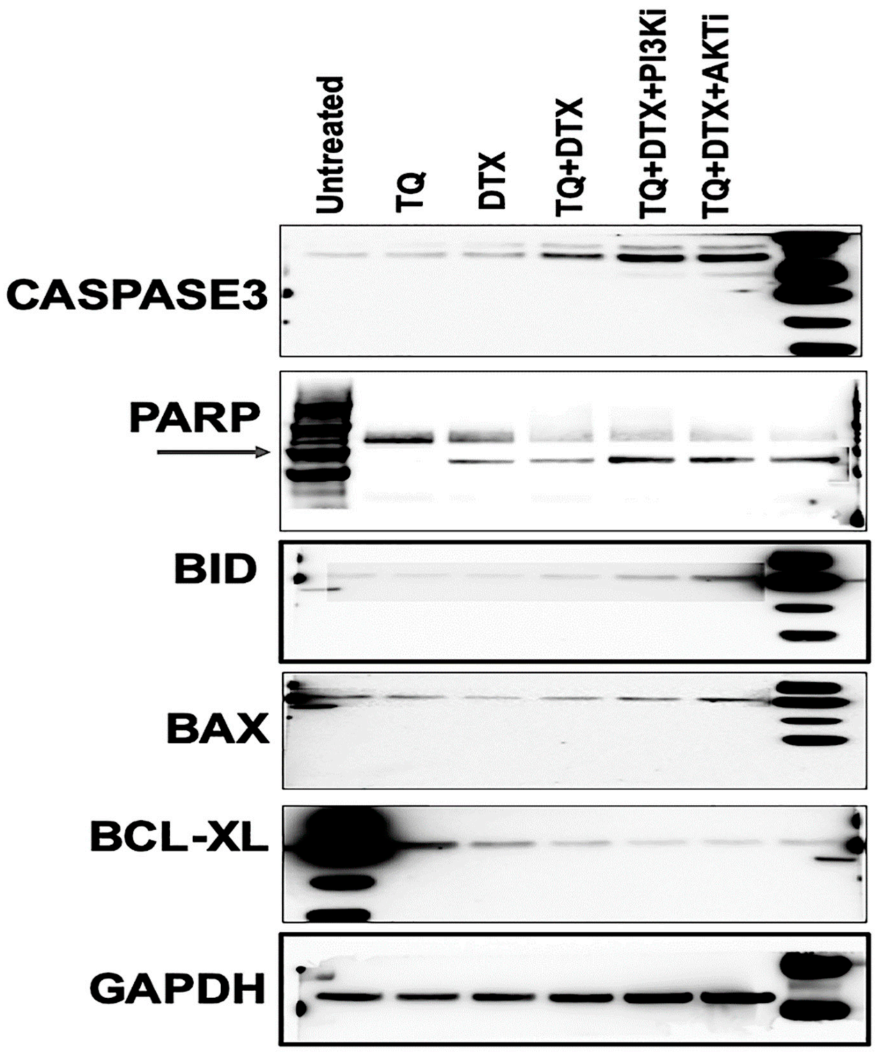

(**ii**)

Figure S1. Western blots and densitometry analysis of pro- and anti-apoptotic markers in DU145 cells. PCa cells were treated with thymoquinone (TQ), docetaxel (DTX), or their combination (TQ+DTX) in the presence or absence of PI3Ki and AKTi inhibitors for 48 h. An equal amount of protein (30 µg) was fractionated on 4–12% polyacrylamide gels and transferred to PVDF membranes. The band intensity of pro-apoptotic (BAX, BID), apoptotic (Caspase3), PARP, and anti-apoptotic (BCL-XL) protein were analyzed by (**i**) densitometry readings/intensity ratio, using ImageJ software (NIH) and were normalized to the corresponding GAPDH value; and (**ii**) immunoblots, respectively. The results were expressed in relative folds change.

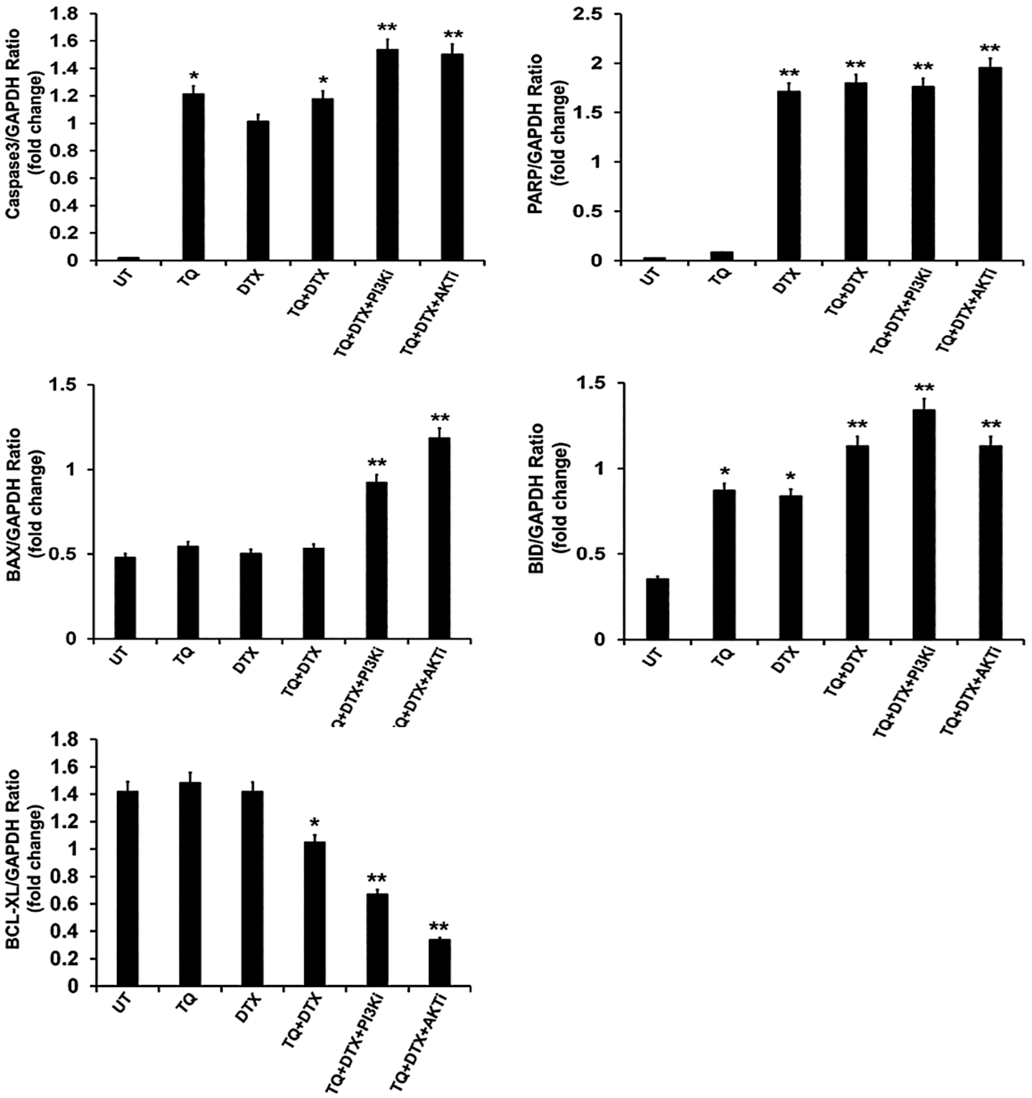

	Caspase 3/GAPDH	PARP/GAPDH	BID/GAPDH	BAX/GAPDH	BCL-XL/GAPDH
UT	0.018959088	0.026889344	0.35210203	0.478020201	1.418465417
TQ	1.210522029	0.083796125	0.87010211	0.546402567	1.483277798
DTX	1.01275483	1.71184061	0.83760012	0.501231012	1.416393662
TQ+DTX	1.17628011	1.795573869	1.130102031	0.532870101	1.048381737
TQ+DTX+PI3Ki	1.534560102	1.760692403	1.340201021	0.922307021	0.671236338
TQ+DTX+AKTi	1.50234501	1.951901201	1.130503103	1.184282021	0.336592843

(**i**) Densitometry Readings/intensity Ratio (C42B)

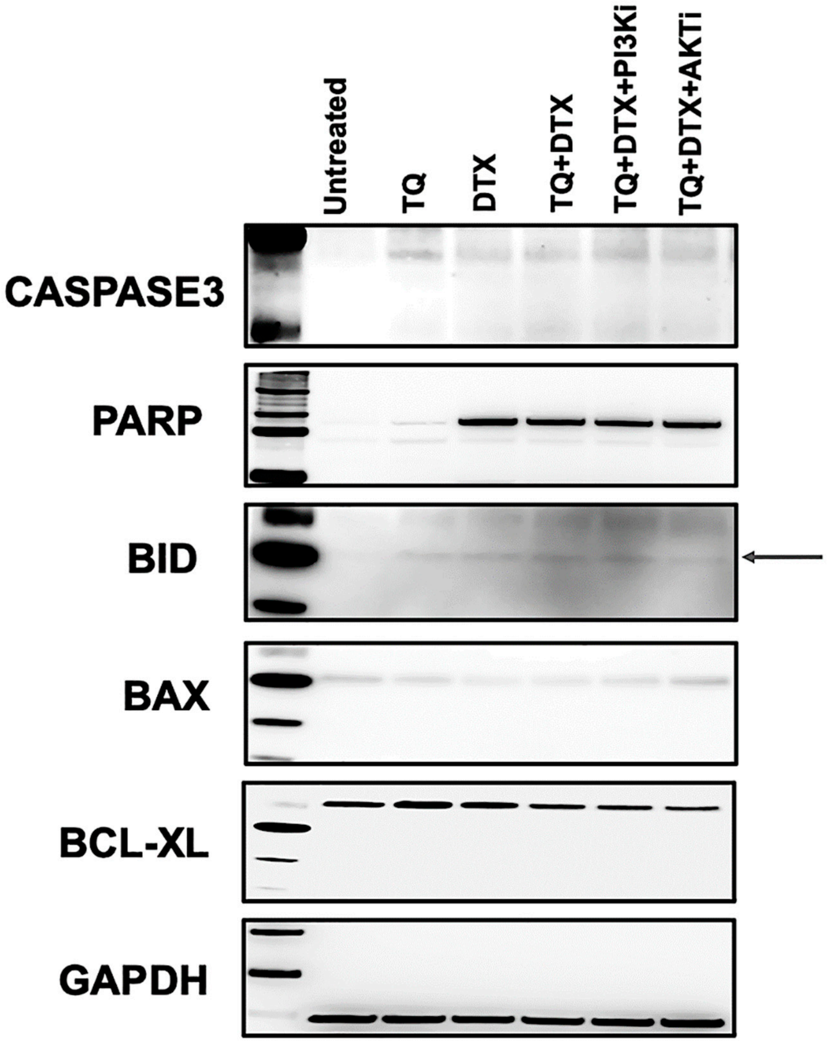

(**ii**)

Figure S2. Western blot and densitometry analysis of pro- and anti-apoptotic markers in C4-2B cells. PCa cells were treated with thymoquinone (TQ), docetaxel (DTX), or their combination (TQ+DTX) in the presence or absence of PI3Ki and AKTi inhibitors for 48 h. An equal amount of protein (30 µg) was fractionated on 4–12% polyacrylamide gels and transferred to PVDF membranes. (**i**) The band intensity of pro-apoptotic (BAX, BID), apoptotic (Caspase3), PARP, and anti-apoptotic (BCL-XL) protein were analyzed by the densitometry readings/intensity ratio using ImageJ software (NIH) and were normalized to the corresponding GAPDH value; and (**ii**) immunoblots, respectively. The results were expressed in relative folds change.
